# Ketogenic diet induces expression of the muscle circadian gene *Slc25a25* via neural pathway that might be involved in muscle thermogenesis

**DOI:** 10.1038/s41598-017-03119-8

**Published:** 2017-06-06

**Authors:** Reiko Nakao, Shigeki Shimba, Katsutaka Oishi

**Affiliations:** 1 Biological Clock Research Group, Biomedical Research Institute, National Institute of Advanced Industrial Science and Technology (AIST), Tsukuba, Ibaraki 305-8566 Japan; 2 Department of Health Science, School of Pharmacy, Nihon University, Funabashi, Chiba 274-8555 Japan; 3 Department of Applied Biological Science, Graduate School of Science and Technology, Tokyo University of Science, Noda, Chiba 278-8510 Japan; 4 Department of Computational and Medical Sciences, Graduate School of Frontier Sciences, The University of Tokyo, Kashiwa, Chiba 277-0882 Japan

## Abstract

We recently found that the mRNA expression of *Slc25a25*, a Ca^2+^-sensitive ATP carrier in the inner mitochondrial membrane, fluctuates in a circadian manner in mouse skeletal muscle. We showed here that the circadian expression of muscle *Slc25a25* was damped in *Clock* mutant, muscle-specific *Bmal1*-deficient, and global *Bmal1*-deficient mice. Furthermore, a ketogenic diet (KD) that induces time-of-day-dependent hypothermia (torpor), induced *Slc25a25* mRNA expression in skeletal muscle. Hypothermia induced by KD did not affect thermogenic genes such as *Sarcolipin* and *Pgc1a* in muscles and *Ucp1* in adipose tissues. Sciatic denervation abolished circadian and KD-induced *Slc25a25* expression, suggesting that the circadian clock regulates muscle *Slc25a25* expression via neural pathways. We measured body temperature (Tb) in sciatic denervated mice fed with KD to determine the functional role of KD-induced *Slc25a25* expression. Sciatic denervation abolished *Slc25a25* expression and augmented KD-induced hypothermia compared with sham-operated mice, but did not affect Tb in mice given a normal diet. These findings suggest that KD feeding induces expression of the muscle circadian gene *Slc25a25* via neural pathways, and that SLC25A25 might be involved in muscle thermogenesis under KD-induced hypothermia in mammals.

## Introduction

Continuous interplay between the circadian clock system and homeostatic mechanisms regulates core body temperature (Tb) within a narrow range in homeothermic animals including mammals, and maintains it independently of the environmental temperature. The circadian rhythm of Tb that increases during activity and declines during rest is governed by the master clock located in the suprachiasmatic nucleus (SCN) of the anterior hypothalamus^1, 2^. Neurons located in the preoptic area of the hypothalamus are believed to contain the central thermostat for the homeostatic control of Tb. It receives and integrates information about peripheral (cutaneous and visceral) and local brain temperatures and provides appropriate command signals to peripheral thermoregulatory effectors that control heat dissipation and production^3–5^.

Adaptive thermogenesis is defined as heat production in response to environmental temperature or diet^6^. Neurons in the preoptic area trigger thermogenesis during exposure to cold by activating descending signals through hypothalamic and medullary sites to drive repetitive contractions of skeletal muscle (shivering)^4^. However, continuous muscle shivering leads to exhaustion and muscle damage. Therefore, non-shivering thermogenesis is activated during chronic cold exposure to sustain heat production through descending output from the hypothalamus to the raphe pallidus nucleus that in turn innervates sympathetic preganglionic neurons^3^. Brown adipose tissue (BAT) is extensively innervated by sympathetic fibers and thus is an important site of non-shivering thermogenesis in most mammals^6^. The important molecule involved in cold-induced thermogenesis in BAT is UCP1, which is a mitochondrial inner-membrane protein that uncouples proton entry from ATP synthesis^6^. Feeding acutely increases metabolic rates in mammals, and thus diet is also a potent regulator of adaptive thermogenesis. On the contrary, when faced with a harsh climate with inadequate food, some mammals periodically turn down their internal thermostat and enter torpor (controlled decrease of the metabolic rate, Tb and physical activity). Torpor is considered a means of survival during periods of low food availability. When food becomes available, such animals can rewarm to return to a normal level of activity^7^. Although the metabolic rate during torpor is strikingly decreased, Tb is regulated above a species-specific minimum by a proportional increase in heat production that compensates for heat loss^8^. The circadian system controls torpor timing that allows some mammals to stay entrained with the light-dark cycle, which facilitates continued foraging^9^. We previously reported that chronic feeding with a ketogenic diet (KD) comprising high fat with low carbohydrate and protein contents induces torpor and significantly decreases core Tb in mice^10^. We also found that KD induces a decrease in Tb particularly late in the dark (active) period, and increases Tb during the light-to-dark transition to a level like that of a normal diet in mice^10^. Several molecules such as PPARα and FGF21 might regulate time-dependent torpor^11^, although we found that both KD and fasting induce hypothermia in FGF21-deficient as well as in wild-type mice^12^. The underlying mechanism that regulates Tb under hypothermia induced by KD remains unknown, although BAT has been considered a major thermogenic organ against exposure to cold.

The circadian oscillator in the SCN is driven by transcription/translation-based autoregulatory feedback loops consisting of the periodic expression of clock genes^2^. Studies of clock genes in mammals have revealed that oscillatory mechanisms function in various peripheral tissues such as the heart, lungs, liver, kidneys, adipose tissues and skeletal muscles, and that they are entrained to the SCN by systemic time cues including neural, humoral and other signals such as feeding and body temperature^2^. Hundreds of circadian clock-controlled genes that regulate a remarkable diversity of biological processes have been identified in peripheral tissues including skeletal muscle using DNA microarray technology^13–16^. The significance of clock and clock-controlled genes in the skeletal muscle has been demonstrated in animal models of molecular clock disruption. For example, muscle force is reduced, mitochondria are dysfunctional and myofilament architecture is disrupted in *Clock* mutant and *Bmal1*-deficient mice^14, 17^. Muscle-specific *Bmal1* knockout (KO) mice have muscle fibrosis^18^ and impaired glucose uptake^19^. These facts suggest that the molecular clock can modify skeletal muscle physiology. Here, we focused on the physiological role of *Slc25a25* among 478 circadian genes in muscle^16^. SLC25A25 is a Ca^2+^-sensitive ATP-Mg^2+^/Pi carrier in the inner membranes of mitochondria that might be associated with thermogenesis in mice^20, 21^. We assessed the circadian regulatory mechanisms of *Slc25a25*, and its putative role in muscle thermogenesis during torpor induced by a KD.

## Results

### Systemic circadian clock regulates rhythmic expression of *Slc25a25* via neural signals

**Figure 1:**
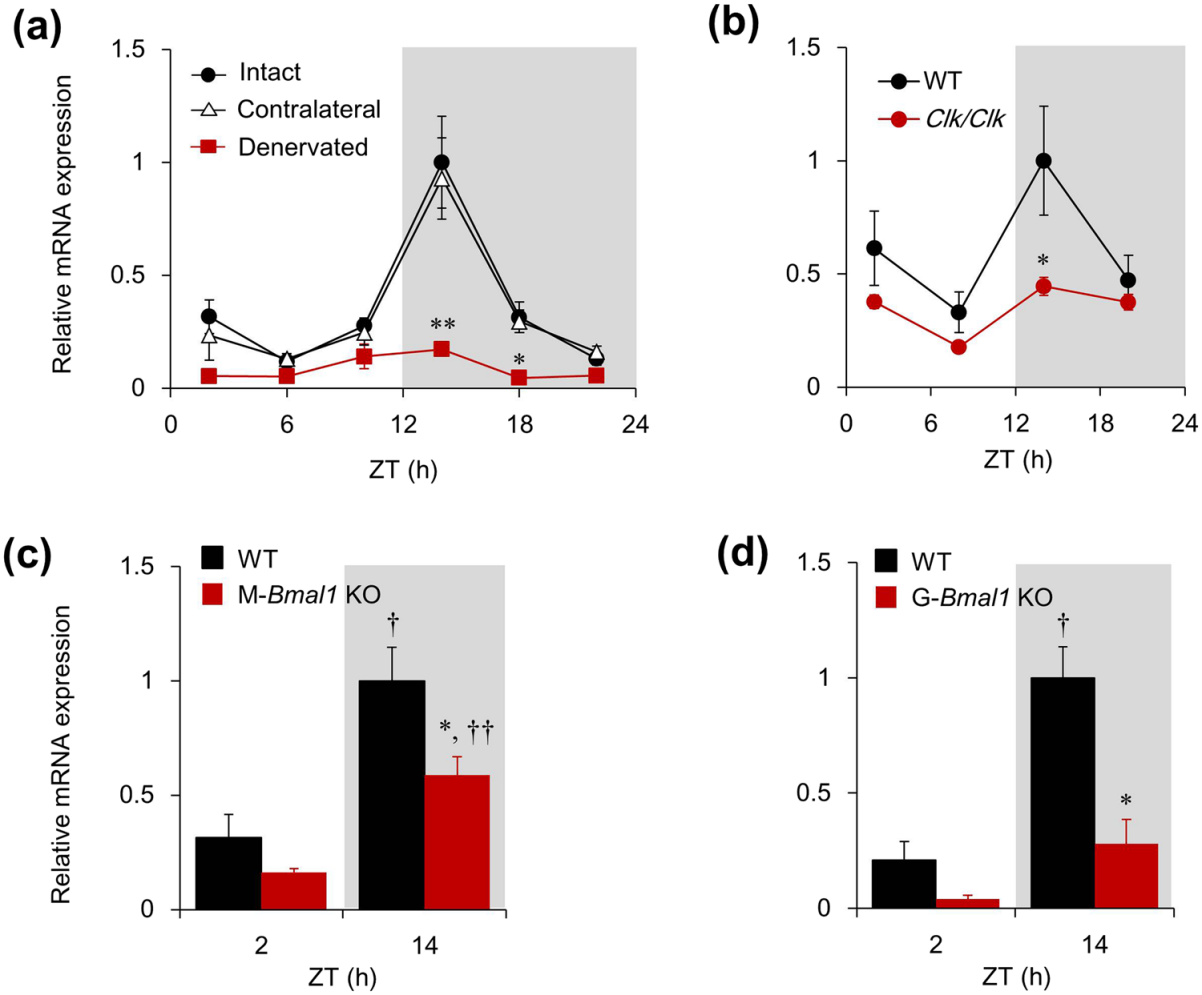
*Slc25a25* is a circadian gene in skeletal muscle. (**a**) Circadian expression of *Slc25a25* in skeletal muscle seven days after sciatic denervation. Contralateral innervated muscles of same mice and muscles of intact mice served as controls. Gray shading indicates dark period. Data are expressed as means ± SEM (n = 5–6 per group). Maximal value for intact mice is expressed as 1.0. ^*^ *P* < 0.05 and ^**^ *P* < 0.01 for intact vs. denervated muscle at corresponding Zeitgeber time (ZT). (**b**) Temporal expression profiles of *Slc25a25* mRNA in skeletal muscle of *Clock* mutant (*Clk/Clk*) mice. Gray shading indicates dark period. Data are expressed as means ± SEM (n = 4–5 per group). ^*^ *P* < 0.05 for WT vs. mutant mice at corresponding ZT. (**c**,**d**) Muscle-specific (M-*Bmal1* KO) or global (G-*Bmal1* KO) *Bmal1* knockout mice. Gray shading indicates dark period. Data are expressed as means ± SEM (n = 4–5 per group). Maximal value for wild-type (WT) mice is expressed as 1.0. ^*^ *P* < 0.05 for WT vs. mutant mice. ^†^ *P* < 0.05 and ^††^ *P* < 0.01 for ZT2 vs. ZT14. Supplemental Tables 2 and 3 show results of statistical analysis.

Among 478 genes in the gastrocnemius muscles of mice that fluctuate in a circadian manner between day and night^16^, 313 lost rhythmicity after sciatic denervation and among these, we investigated *Slc25a25* that encodes a Ca^2+^-sensitive ATP-Mg^2+^/Pi carrier in the inner membranes of mitochondria^20^. We initially assessed the effect of sciatic denervation on the circadian expression of *Slc25a25* mRNA in mouse gastrocnemius muscles. The mRNA expression of *Slc25a25* fluctuated in a circadian manner that peaked at zeitgeber time (ZT) 14 in intact and contralateral skeletal muscle (both *P* < 0.001; one-way ANOVA) (Fig. 1a). The circadian amplitude of *Slc25a25* expression was decreased by 85% in the gastrocnemius muscle of mice with sciatic denervation relative to that in intact muscle (Fig. 1a), although the rhythmic expression of *Slc25a25* was retained (*P* = 0.003; one-way ANOVA). We investigated the temporal expression profiles of *Slc25a25* in homozygous *Clock* mutant (*Clk/Clk*), muscle-specific *Bmal1* (M-*Bmal1*) KO, and global *Bmal1* (G-*Bmal1*) KO mice to assess whether or not the molecular clock is involved in the circadian regulation of *Slc25a25* expression. The circadian amplitude of *Slc25a25* mRNA expression was decreased by 60% in the skeletal muscle of *Clk/Clk* compared with that of WT mice (Fig. 1b), although its mRNA levels remained rhythmic (*P* < 0.001; one-way ANOVA). The muscle-specific deletion of *Bmal1* slightly decreased the peak level of *Slc25a25* expression, but day/night expression was essentially retained (WT, *P* = 0.016; M-*Bmal1* KO, *P* = 0.002; *t*-test) (Fig. 1c). The peak expression level of *Slc25a25* was significantly lower in G-*Bmal1* KO, than in WT mice, and day/night oscillation was abolished in G-*Bmal1* KO (WT, *P* < 0.001; G-*Bmal1* KO, *P* = 0.087; *t*-test; Fig. 1d).

### Ketogenic diet induces *Slc25a25* mRNA expression in skeletal muscle, but not in brown and white adipose tissues

**Figure 2:**
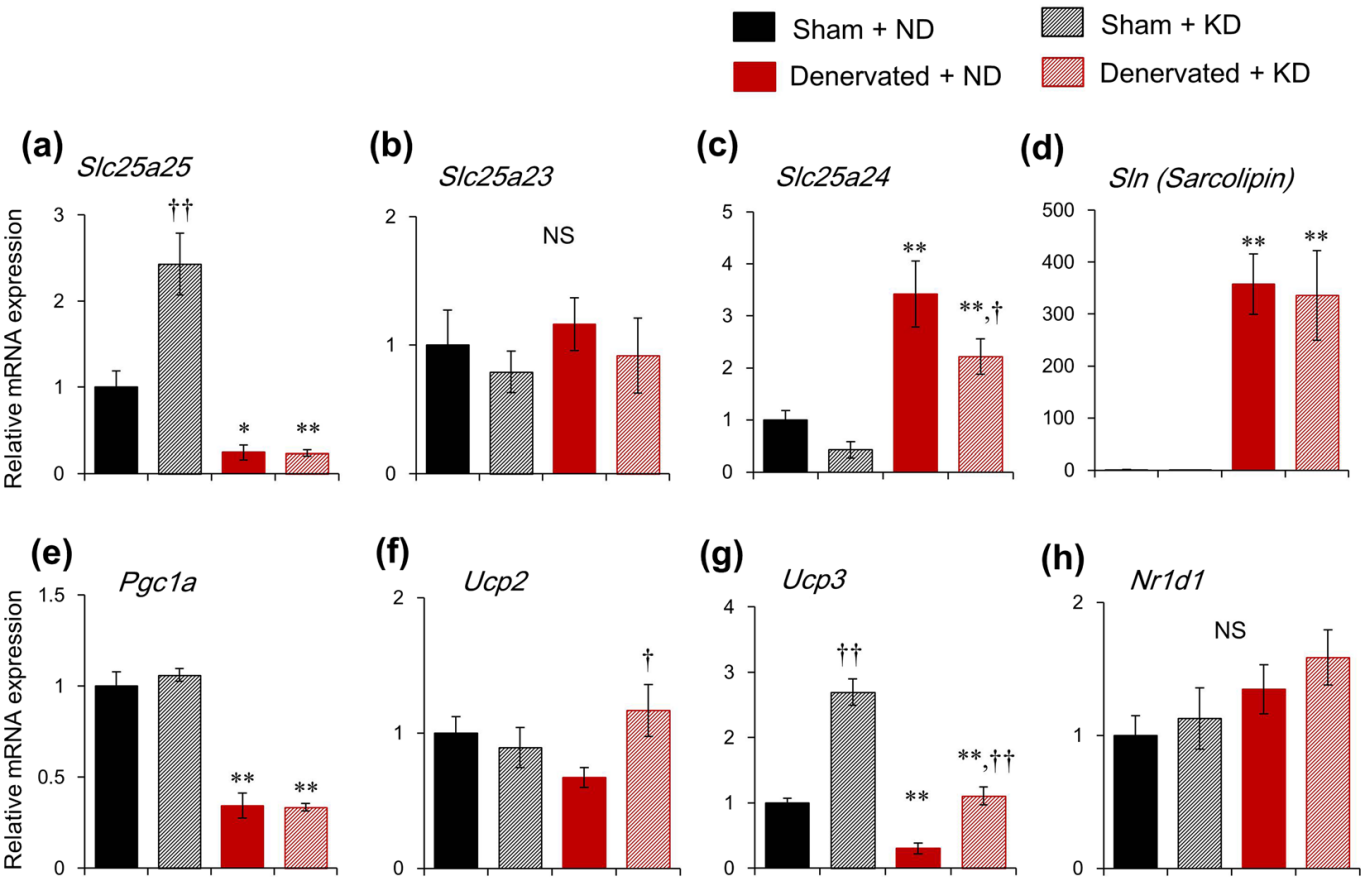
Prolonged ketogenic diet feeding upregulates *Slc25a25* expression in skeletal muscle. Messenger RNA expression of thermogenic gene in skeletal muscle of mice fed with ketogenic (KD) or normal (ND) diets for seven days starting from 10 days after sciatic denervation or sham-operation. Data are expressed as means ± SEM (n = 5 per group). Value for sham-operated mice fed with ND is expressed as 1.0. ^*^ *P* < 0.05 and ^**^ *P* < 0.01 for sham-operated vs. denervated. ^†^ *P* < 0.05 and ^††^ *P* < 0.01 for ND vs. KD. Supplemental Table 4 shows results of statistical analysis.

A previous study has shown that SLC25A25 might contribute to a thermogenic pathway in mice with defective BAT thermogenesis, because *Slc25a25* mRNA expression is induced during adaptation to cold stress in the skeletal muscle of *Ucp1* KO mice^21^. On the other hand, normal Tb was maintained in global *Slc25a25* KO mice after acute exposure to cold stress, suggesting that SLC25A25 is not essential for the regulation of Tb during exposure to cold^21^. We investigated the mRNA expression of *Slc25a25* in sciatic denervated and sham-operated mice after seven days on a ketogenic diet (KD) that induces time-of-day-dependent hypothermia (torpor)^10^. In addition to *Slc25a25*, we also measured the mRNA expression of other thermogenic genes such as those for *Sarcolipin* (*Sln*), *Pgc1a*, *Ucp2*, and *Ucp3*. Sarcolipin regulates sarcoplasmic reticulum Ca^2+^-ATPase (SERCA) and it is involved in non-shivering muscular thermogenesis during cold stress^22^. The thermogenic molecule PGC1α functions as a transcriptional co-activator of PPARγ that modulates the expression of *Ucp1* and thermogenesis in brown fat^23^. The KD upregulated mRNA expression of *Slc25a25* and *Ucp3* by 2.4-fold and 2.7-fold, respectively, in muscle from sham-operated mice compared with mice fed with a normal diet (ND) (Fig. 2a,g). However, the KD did not induce the mRNA expression of *Slc25a25* isoforms such as *Slc25a23* and *Slc25a24* (Fig. 2b,c). The KD also did not affect the mRNA expression of *Sln*, *Pgc1a* and *Ucp2* in muscle from sham-operated mice (Fig. 2d–f). On the other hand, sciatic denervation gene-specifically affected the mRNA expression of these genes; it significantly decreased the mRNA expression levels of *Slc25a25*, *Pgc1a*, and *Ucp3*, but increased those of *Slc25a24* and *Sln*. The expression levels of *Sln* were increased about 400-fold by denervation independently of the diet (Fig. 2d). Among the thermogenic genes measured herein, KD increased *Slc25a25* and *Ucp3* expression in sham-operated muscle, and only *Slc25a25* abolished the response to KD by denervation. The mRNA expression levels of *Nr1d1* that inhibits the thermogenic activity of BAT^24^ were not affected by either the KD or denervation (Fig. 2h).

**Figure 3:**
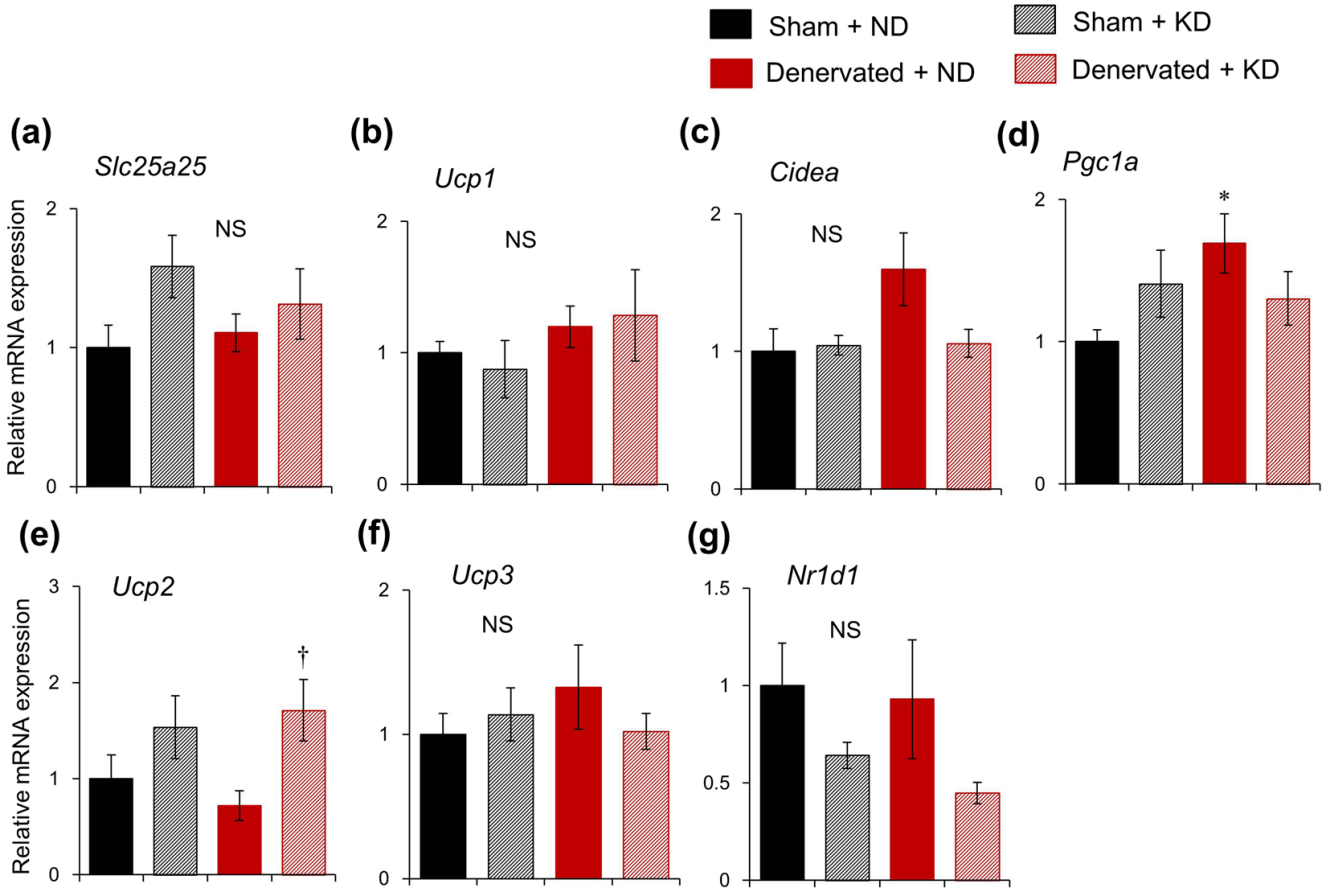
Messenger RNA expression of thermogenic gene in brown adipose tissue. Prolonged ketogenic diet (KD) feeding minimally affects thermogenic gene expression in brown adipose tissues of mice fed with KD or normal diet (ND) for seven days starting at 10 days after sciatic denervation or sham-operation. Data are expressed as means ± SEM (n = 5 per group). Value for sham-operated mice fed with ND is expressed as 1.0. ^*^ *P* < 0.05 for sham-operated vs. denervated. ^†^ *P* < 0.05 for ND vs. KD. Supplemental Table 4 shows results of statistical analysis.

Brown adipose fat is thought to be the primary contributor to thermogenesis through the function of UCP1^6^. We examined the effects of the KD and sciatic denervation on the expression of thermogenic genes in BAT, and found that neither the KD, nor sciatic denervation significantly affected *Slc25a25* expression (Fig. 3a). Furthermore, the mRNA expression of the thermogenic genes, *Ucp1*, *Cidea*, *Ucp3* and *Nr1d1* did not significantly differ among all groups (Fig. 3b,c,f,g). Sciatic denervation slightly upregulated *Pgc1a* mRNA expression, whereas the KD did not (Fig. 3d), although it increased *Ucp2* expression in denervated mice (Fig. 3e).

**Figure 4:**
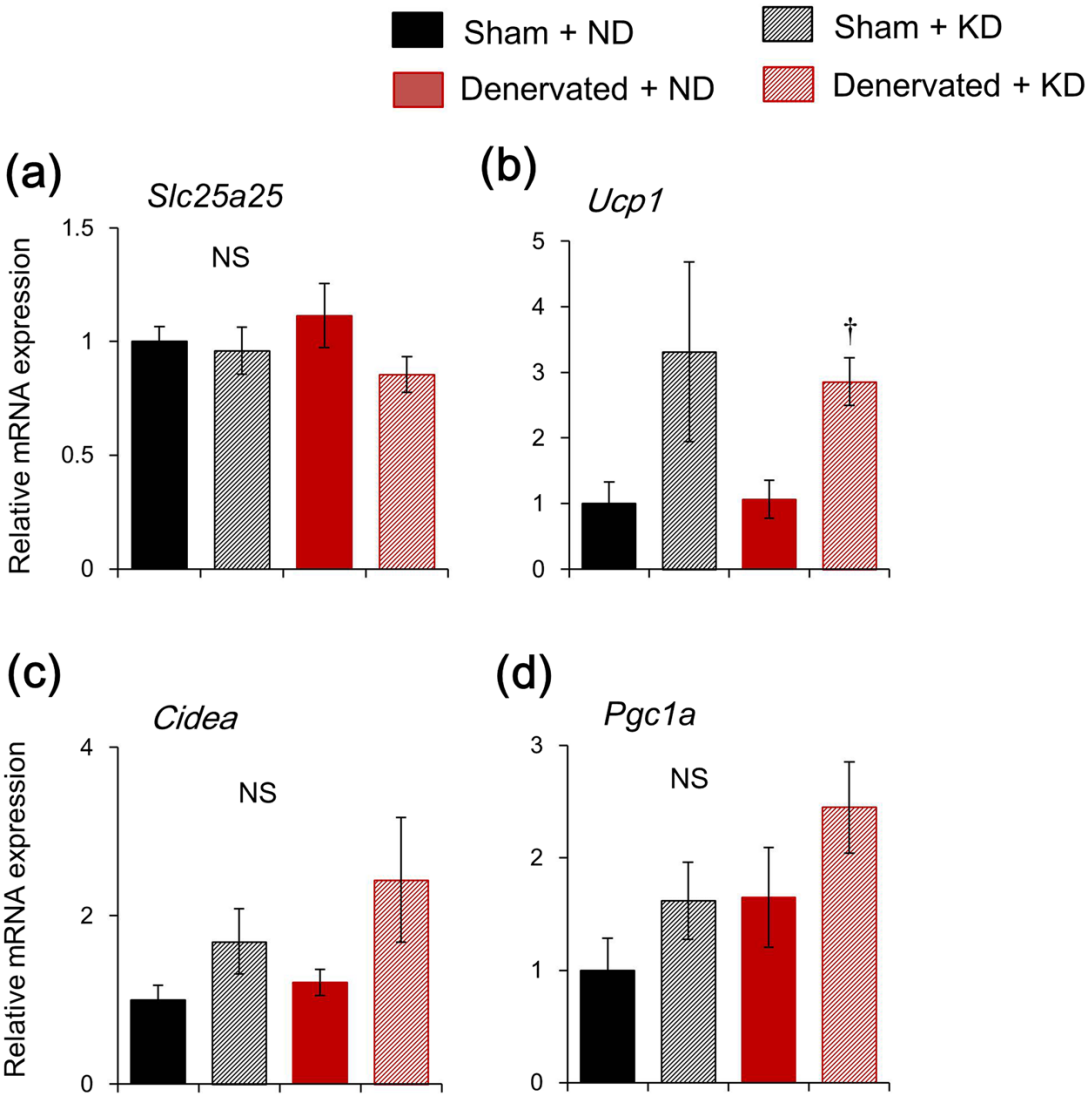
Messenger RNA expression of thermogenic genes in white adipose tissue. Prolonged ketogenic diet (KD) feeding minimally affects thermogenic gene expression in white adipose tissues of mice fed with KD or normal diet (ND) for seven days starting at 10 days after sciatic denervation or sham-operation. Data are expressed as means ± SEM (n = 5 per group). Value for sham-operated mice fed with ND is expressed as 1.0. ^†^ *P* < 0.05 for ND vs. KD. Supplemental Table 4 shows results of statistical analysis.

We evaluated the mRNA expression of thermogenic genes in white adipose tissue (WAT), because beige cells in WAT are involved in adaptation to a chronically cold environment^25–28^. Levels of *Slc25a25*, *Cidea* and *Pgc1a* gene expression did not significantly differ among all groups (Fig. 4a,c,d). The KD slightly induced the mRNA expression of *Ucp1* in denervated and sham-operated mice (Fig. 4b).

### Sciatic denervation augments hypothermia induced by a ketogenic diet

**Figure 5:**
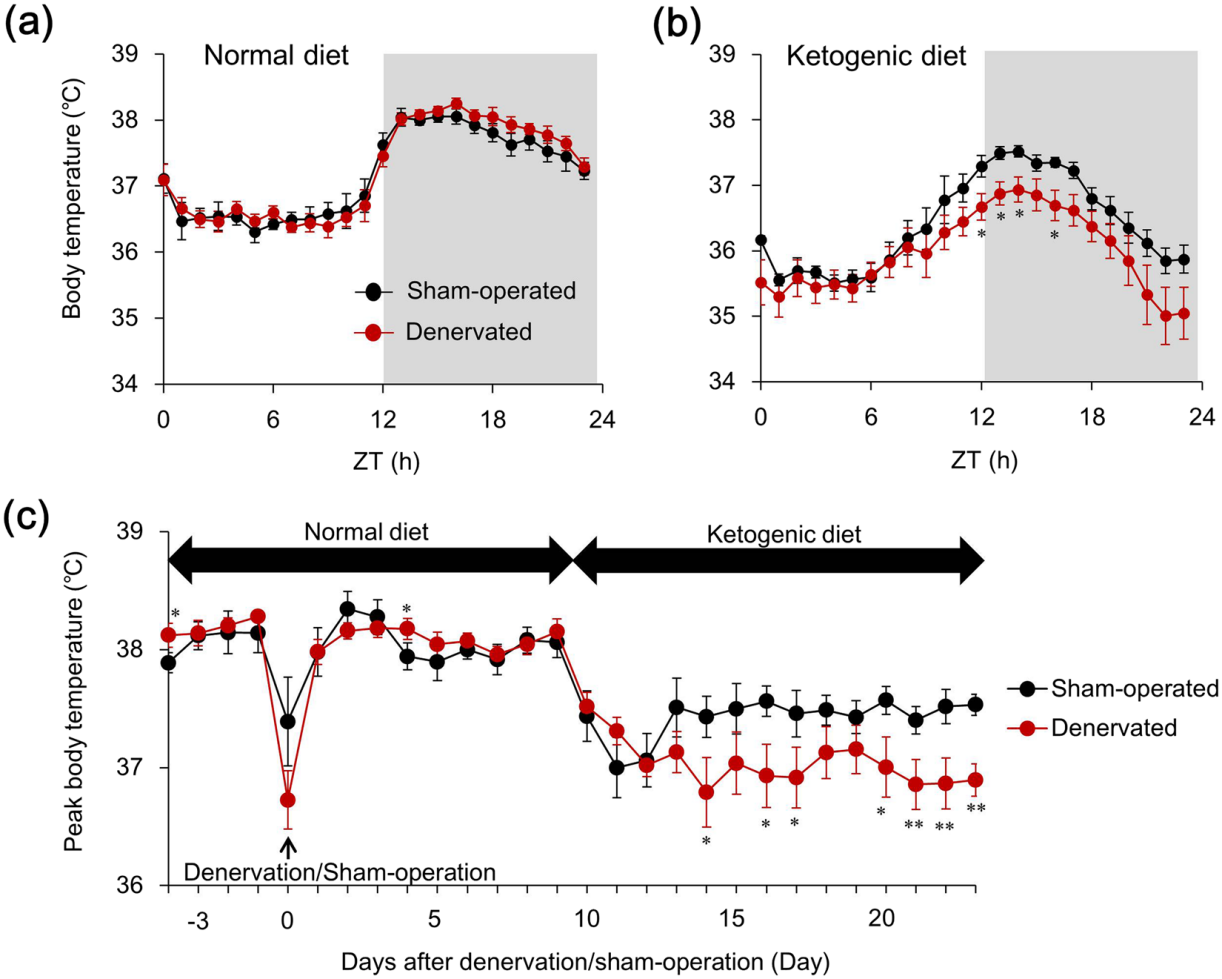
Bilateral sciatic denervation exacerbates hypothermia during prolonged ketogenic diet feeding. Core body temperature rhythms for 24 h in mice fed with ketogenic (KD) or normal (ND) diets for two weeks starting at 10 days after sham operation or bilateral sciatic nerve transection. Hourly averaged values of body temperature on the day before starting (**a**) and after two weeks (**b**) on ketogenic diet. Gray shading indicates dark period. (**c**) Peak values of body temperature during experimental period. Body temperatures were averaged every two hours from ZT13 to ZT15. Data are expressed as means ± SEM (n = 5–7 per group). ^*^ *P* < 0.05 and ^**^ *P* < 0.01 for sham-operated vs. denervated. ZT, zeitgeber time. Supplemental Tables 5 and 6 show results of statistical analysis.

We assessed the effect of the KD on circadian Tb rhythm in sciatic denervated and sham-operated mice to determine the functional role of the skeletal muscle-specific induction of *Slc25a25* mRNA expression in mice fed with the KD. The circadian fluctuation of Tb that peaked during the early night was identical between denervated and sham-operated groups fed with the ND (Fig. 5a). The KD obviously reduced Tb during the latter half of the dark period as described^10^ in both denervated and sham-operated mice (Supplemental Fig. 1). Notably, Tb was significantly lower during the early night in denervated, than in sham-operated mice fed with the KD (Fig. 5b), although denervation did not affect the Tb rhythm in mice fed with a normal diet (Fig. 5a). We compared the effect of KD on peak Tb between denervated and sham-operated mice, and found that sciatic denervation obviously augmented KD-induced hypothermia (Fig. 5c). Fourteen days of feeding with the KD decreased peak Tb from 38.1 ± 0.13 °C (on the day before KD feeding) to 37.5 ± 0.09 °C in sham-operated, and from 38.2 ± 0.11 °C to 36.9 ± 0.14 °C in denervated mice (Fig. 5c). These results indicate that skeletal muscle plays an important role in maintaining Tb while under feeding with a KD, although it does not seem essential under normal diet feeding.

### Aging affects thermogenic gene expression in skeletal muscle

**Figure 6:**
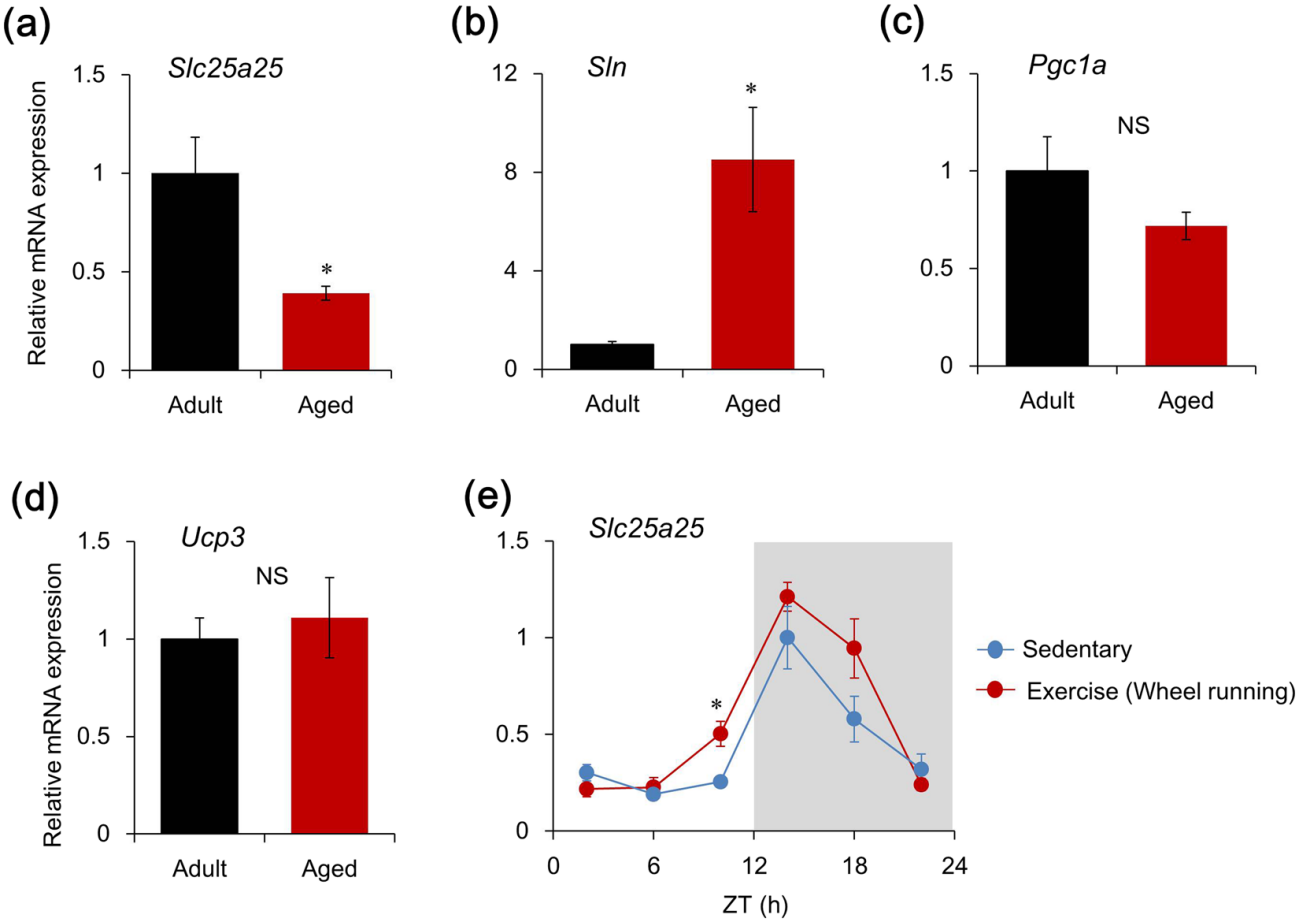
Aging gene-dependently affects thermogenic gene expression. (**a**–**d**) Messenger RNA expression of thermogenic gene in skeletal muscles of adult and aged mice (aged 7–8 and 23–24 months, respectively). Data are expressed as means ± SEM (n = 6–10 per group). Maximal value for adult mice is expressed as 1.0. ^*^ *P* < 0.05 for adult vs. aged mice. (**e**) Effects of voluntary running on *Slc25a25* mRNA expression in skeletal muscle. Mice were individually housed in cages with or without (to mimic sedentary conditions) running-wheels for four weeks. Gray shading indicates dark period. Data are shown as means ± SEM (n = 4–5). Maximal value for sedentary mice is expressed as 1.0. ^*^ *P* < 0.05 for sedentary vs. running-wheel. Supplemental Tables 7 and 8 show results of statistical analysis.

We assessed the thermogenic gene expression in skeletal muscle to determine the functional role of *Slc25a25* on the decrease in Tb induced by aging. The levels of *Slc25a25* and *Sln* mRNA expression were significantly decreased and increased, respectively, in the skeletal muscle of aged mice (Fig. 6a and b). Levels of *Pgc1a* and *Ucp3* expression were essentially identical between adult and aged mice (Fig. 6c and d). We also examined the effect of chronic exercise on *Slc25a25* expression in skeletal muscle. We found that four weeks of voluntary wheel running^29^ increased the mRNA expression of *Slc25a25* (Fig. 6e), but not of *Ucp3* (Supplemental Fig. 2) in skeletal muscle compared with that in sedentary mice.

## Discussion

The present study aimed to elucidate the functional role of circadian *Slc25a25* expression in mouse skeletal muscle. We found that the circadian amplitude of *Slc25a25* expression was decreased by 85% in denervated, compared with intact muscle. The circadian amplitude of muscle *Slc25a25* expression was decreased by 60%, 38% and 70% in *Clk/Clk*, M-*Bmal1* KO, and G-*Bmal1* KO mice, respectively. These findings suggest that the circadian expression of *Slc25a25* is largely dependent on neural signals in a molecular clock-dependent manner. A previous study found that SLC25A25 might contribute to a thermogenic pathway in mice with defective BAT thermogenesis, because *Slc25a25* mRNA expression was induced during adaptation to cold stress in the skeletal muscle of *Ucp1* KO mice^21^. On the other hand, that study also found that Tb remained normal in global *Slc25a25* KO mice under acute exposure to cold stress, suggesting that SLC25A25 is not essential for Tb regulation during cold exposure^21^. The present study evaluated the functional role of *Slc25a25* on adaptive thermogenesis in mice fed with a KD, which induces time-of-day dependent hypothermia (torpor). The KD significantly increased the mRNA expression of *Slc25a25* in muscle. This was probably muscle-specific and governed by neural signals, because the KD did not induce *Slc25a25* mRNA in other tissues such as BAT, WAT and the liver (Figs 3a, 4a, and Supplemental Fig. 3), and sciatic denervation abolished KD-induced mRNA expression. The mRNA expression of typical thermogenic molecules such as *Ucp1* in BAT and WAT, and of *Sln* and *Pgc1a* in skeletal muscles was not affected by the KD. To evaluate the functional role of the skeletal muscle-specific induction of *Slc25a25* mRNA expression in mice fed with the KD, we investigated the effects of the KD on circadian Tb rhythms in sciatic denervated and sham-operated mice. The Tb was significantly lower in sciatic denervated mice fed with the KD compared with sham-operated control mice, whereas it remained essentially identical between denervated and sham-operated mice fed with the ND, suggesting that muscle thermogenesis is involved in the maintenance of core Tb under KD feeding. The present findings suggest that both the central clock and energy homeostasis regulate muscle *Slc25a25* expression via neural pathways, and that SLC25A25 may be involved in muscle thermogenesis under hypothermia induced by KD in mammals.

We assessed the temporal expression profiles of *Slc25a25* and the typical clock gene, *Per2*, in C2C12 myotubes to determine the involvement of peripheral clocks in the rhythmic expression of *Slc25a25* mRNA. However, we did not find rhythmic *Slc25a25* expression in C2C12 myotubes, although the circadian expression of *Per2* was robust (Supplemental Fig. 4). These findings suggest that molecular clock components are involved in the circadian expression of *Slc25a25* in a systemic manner. On the other hand, day/night fluctuation of *Slc25a25* expression was slight, but significantly retained in denervated (*P* < 0.001; one-way ANOVA), *Clk/Clk* (*P* < 0.001; one-way ANOVA), or M-*Bmal1* KO mice (*P* = 0.002; *t*-test), but not in G-*Bmal1* KO mice (*P* = 0.087; *t*-test). All experiments in the present study proceeded under light-dark cycles. Day/night locomotor activity rhythm was evident in denervated, *Clk/Clk*, and M-*Bmal1* KO mice as well as WT animals, the result of a direct masking effect (Supplemental Fig. 5). Day/night behavioral and physiological rhythms including feeding and body temperature might, at least in part, be involved in the circadian regulation of *Slc25a25* expression in muscle.

Muscle mRNA levels of *Slc25a25* robustly fluctuated in a circadian manner that peaked at the day-to-night transition and were positively regulated by CLOCK and BMAL1 in a systemic manner through a neural pathway. However, the mRNA levels of *Slc25a25* remained almost constant throughout the day in the liver, and upregulated in *Clk/Clk* mice (Supplemental Fig. 6a). These findings suggest that *Slc25a25* transcription is tissue-specifically regulated and that muscle SLC25A25 plays an important role during the active onset.

We exposed mice to time-restricted feeding (diet available for 8 h during either daytime or nighttime) for one week^30^ to determine whether the circadian feeding schedule is involved in the circadian regulation of muscle *Slc25a25* expression. This feeding schedule did not affect the circadian phase of *Slc25a25* mRNA expression, which peaked at ZT14 in skeletal muscle (Supplemental Fig. 7a). In contrast, the reversed feeding schedule obviously affected the temporal expression profile of *Slc25a25* mRNA in both the liver and WAT of mice (Supplemental Fig. 7b,c). The acrophase of mRNA expression in the liver and WAT corresponded to the feeding phase. These results suggest that feeding-derived signals are important to enforce the rhythmicity of *Slc25a25* expression in the liver and WAT, but not in skeletal muscle. Feeding schedules do not affect the central clock in the SCN^31^. Therefore, neural signals from the SCN clock might be involved in the circadian regulation of *Slc25a25* expression in skeletal muscle.

We analyzed the effects of a *Clock* gene mutation on *Slc25a25* expression induced by the KD (Supplemental Fig. 8). We found that the KD-induced *Slc25a25* expression in *Clk/Clk* mice, although to a lesser extent than in WT mice. These findings suggest that the molecular clock is dispensable, but partly involved in the KD-induced *Slc25a25* expression in muscle.

We did not identify an effect of KD feeding on *Ucp1* expression in either muscle or BAT, although previous studies have demonstrated KD-induced *Ucp1* expression in BAT^32, 33^. We analyzed the effects of short-term (7 days) KD feeding, whereas Kennedy *et al*. and Srivastava *et al*. assessed the long-term effects (5 and 4 weeks, respectively)^32, 33^. The effects of KD on metabolic tissues might vary dependently on the experimental period.

The present study found that sciatic denervation significantly decreased nighttime Tb in mice fed with KD, but did not affect Tb in mice fed with a normal diet. Sciatic denervation did not affect starvation-induced endocrine systems since the KD similarly increased plasma FGF21 concentrations in both denervated and sham-operated mice (Supplemental Fig. 9). Importantly, expression levels of the thermogenic genes, *Ucp1* and *Pgc1a* in BAT and WAT were insensitive to the KD, although Tb was extremely decreased. These observations suggest that muscle thermogenesis plays an important role in maintaining Tb under KD feeding. Muscle thermogenesis has been explained as burst contractions of skeletal muscle (shivering) in an immediate response to acute cold stress^34, 35^. However, shivering is not a long-term continuous thermogenic response but rather a transient thermogenic response to maintain the Tb for a few hours^36^. The KD did not affect the mRNA expression of *Sln* in muscle, although *Sln* is thought to be involved in non-shivering muscular thermogenesis during cold stress^22^. Therefore, SLN does not seem to be a critical contributor to the maintenance of Tb under a KD. Denervation remarkably increased the levels of *Sln* mRNA expression in muscle. These findings suggest that neural signals negatively and positively regulate the expression of *Sln* and of *Slc25a25*, respectively, in muscle.

Circadian amplitude and mean Tb decreases in elderly persons^37–39^. Changes in Tb rhythms associated with aging are frequently associated with a reduction in nighttime sleep quality^40^ and cerebral blood flow^39^. Levels of *Slc25a25* mRNA expression were significantly decreased in the skeletal muscle of aged, compared with adult mice (Fig. 6a), whereas the levels of *Sln* mRNA expression were significantly increased (Fig. 6b). Several studies have found denervation in aging muscles^41^, including a progressive reduction in the number of motoneurons in the spinal cord beginning around the age of 60 years^42^, a loss of motoneurons in the periphery^43, 44^, degeneration of neuromuscular junctions^45^ and loss of motor units^46^. Aging-induced denervation might cause the downregulation of *Slc25a25* in elderly persons, subsequent to a lower Tb. Meanwhile, *Sln* mRNA expression was significantly upregulated in aged muscle of mice (Fig. 6b). We speculated that aging-induced denervation increases *Sln* mRNA expression, and that the thermogenic function of *Sln* seems independent of aging-induced hypothermia. We also examined the effect of chronic exercise on *Slc25a25* expression in skeletal muscle, because exercise is considered an effective approach to improve denervation in elderly persons^47^. We found that four weeks of voluntary wheel running^29^ increased *Slc25a25* mRNA expression in the skeletal muscles of mice (Fig. 6e). Thus, elderly persons should maintain muscle thermogenic activity to retain *Slc25a25* expression and prevent an age-related decrease in Tb. Regular exercise might be a useful approach to maintain muscle thermogenesis through induction of the *Slc25a25* gene.

We could not evaluate the effects of aging on KD-induced *Slc25a25* expression in mice because such experiments are very time-consuming. Further studies are needed to understand the relationships between aging and decreased *Slc25a25* expression, including the effect of KD feeding and sciatic denervation.

**Figure 7:**
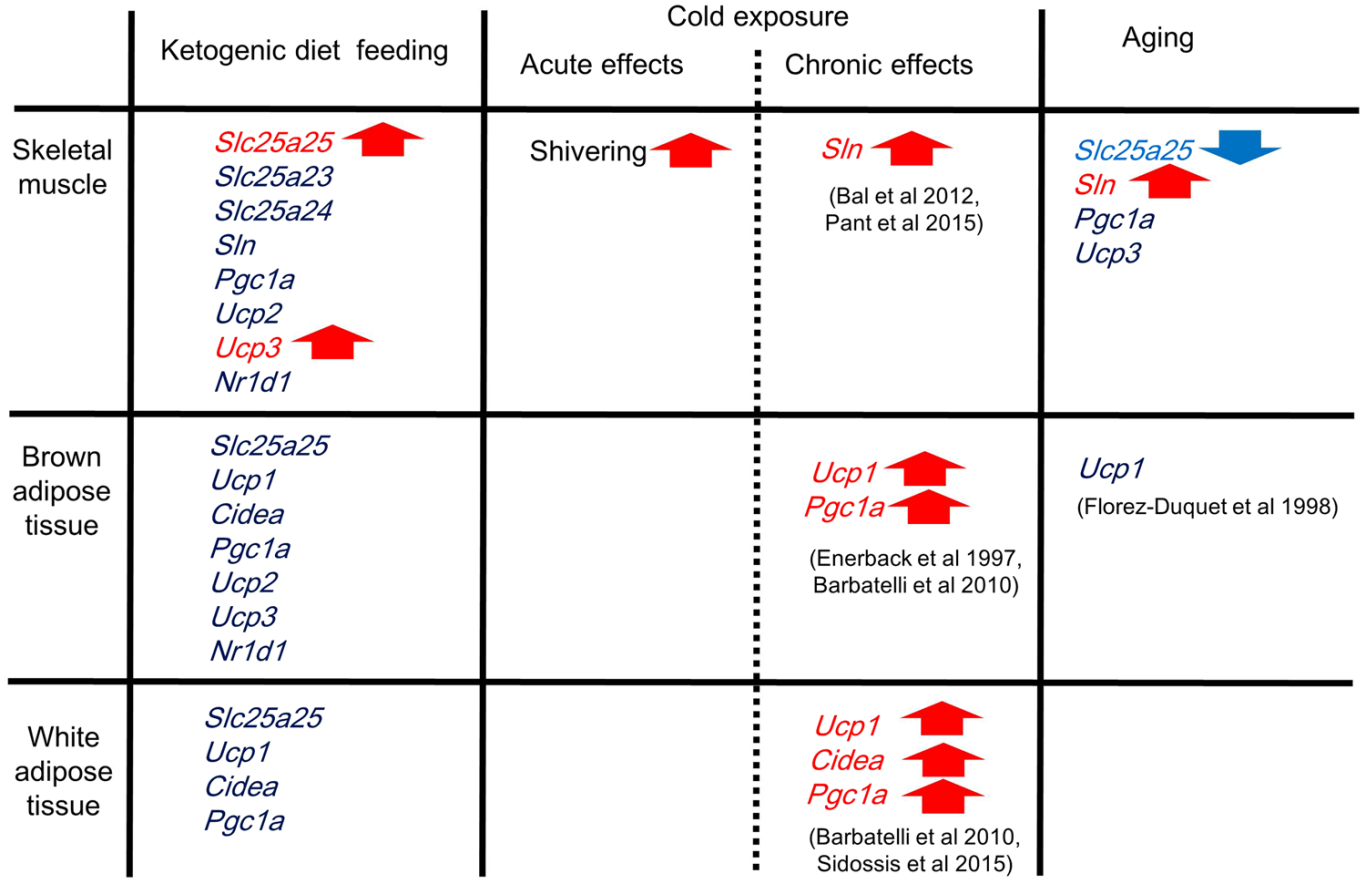
Summary of present study. Mechanism of muscle thermogenesis starts with shivering as first defense against exposure to cold. Thereafter, *Sln* ^22, 50^ and *Ucp1* ^51, 52^ are respectively driven to elicit non-shivering thermogenesis in skeletal muscle and BAT. Exposure to cold also induces emergence of brown adipocytes in WAT by increasing expression of *Ucp1*, *Cidea*, and *Pgc1a* genes^52, 53^. Meanwhile, the present study found induced circadian *Slc25a25* gene expression in skeletal muscle, but not in BAT and WAT of mice fed with ketogenic diet (KD), which induces torpor. Messenger RNA expression of thermogenesis-related genes such as *Ucp1* was not affected by KD in BAT and WAT. Sciatic denervation abolished *Slc25a25* expression and augmented KD-induced hypothermia. These results suggest that skeletal muscle is involved in thermogenesis during KD feeding, and that *Slc25a25* and *Ucp3* are candidates for muscle thermogenesis. Less *Slc25a25* was expressed in skeletal muscle of aged, compared with adult mice, whereas *Ucp1* expression was not altered by aging at thermoneutrality^54^. SLC25A25 might help to maintain Tb in aged mammals.

The present findings suggest that chronic KD feeding induces expression of the circadian gene, *Slc25a25*, via a neural pathway in muscle. Moreover, muscle non-shivering thermogenesis seemed important for maintaining Tb under KD feeding, and *Slc25a25* and *Ucp3* might be involved in muscle thermogenesis (Fig. 7). This is the first report to suggest that thermogenesis derived from skeletal muscle is involved in the maintenance of core Tb under metabolic hypothermia. Sciatic denervation affects the expression of many metabolic genes as we have previously shown^16^. We believe that several genes are associated with muscle thermogenesis under metabolic hypothermia such as ketogenic conditions. In fact, the expression profiles of *Ucp3* and *Slc25a25* in skeletal muscle in response to the KD and sciatic denervation were similar. Further studies are needed to uncover the molecular mechanism of muscle thermogenesis under metabolic hypothermia.

## Methods

### Animal care and surgical procedures

All animal experiments proceeded according to the guidelines for animal experiments of the National Institute of Advanced Industrial Science and Technology (AIST). The Animal Care and Use Committees at AIST approved all the experimental protocols described herein (Permission # 2016-166).

Circadian expression of the *Slc25a25* gene was measured in C57BL/6J mice seven days after sciatic denervation. The contralateral innervated (sham-operated) muscles of the same animals and the muscles of intact animals served as controls, as described^16^. Homozygous *Clock* mutant mice were generated as described^48^. Global *Bmal1* KO mice, muscle-specific *Bmal1* KO mice and their control littermates generated as described^49^ were housed with access to a standard diet (CE2: CLEA Japan Inc., Tokyo, Japan) and water *ad libitum* under a 12 h light-12 h dark cycle (LD12:12); lights on at Zeitgeber time (ZT) 0 and lights off at ZT12. After sacrifice at the indicated times, the gastrocnemius muscles were dissected.

We assessed the effect of KD feeding on the expression of thermogenic genes and Tb in nine week-old male Jcl:ICR mice (Japan SLC Inc., Shizuoka, Japan) that were housed and fed *ad libitum* for two weeks under a 12 h light-12 h dark cycle. The mice were randomly assigned to receive muscle-denervation or a sham operation. The sciatic nerve was bilaterally transected under anesthesia. Sham-operated mice underwent identical dissection without transection. Ten days later, the denervated and sham-operated mice were each divided into two experimental groups and fed with either the AIN-93G (Oriental Yeast Co. Ltd., Tokyo, Japan) normal diet (ND) or the modified AIN-93G ketogenic (KD) diet (73.9% fat, 8.3% protein and 0.73% carbohydrate, w/w; Oriental Yeast Co. Ltd.) for two weeks. The proportions of calories derived from fat, carbohydrate and protein were ND: 12.6%, 58.3% and 29.3%; KD: 94.8%, 0.1% and 4.8%, respectively. The mice were sacrificed at ZT14 and the gastrocnemius muscle, white (WAT) and brown (BAT) adipose tissues were dissected, weighed and frozen in liquid nitrogen.

Mice aged 7–8 (adult) and 23–24 months (aged) were sacrificed to evaluate the effects of aging on thermogenic gene expression in the gastrocnemius muscles.

Seven-week-old male mice were individually housed in cages without running-wheels to mimic sedentary conditions or with running-wheels for four weeks to evaluate the effects of chronic exercise on *Slc25a25* mRNA expression in the gastrocnemius muscles.

### Real-time reverse transcription-polymerase chain reaction (RT-PCR)

Total RNA was extracted using guanidinium thiocyanate followed by RNAiso Plus (Takara Bio Inc., Otsu, Japan). Single-stranded cDNA was synthesized using PrimeScript™ RT reagent kits with gDNA Eraser (Takara Bio). Real-time RT-PCR proceeded using SYBR^®^ Premix Ex Taq™ II (Takara Bio) and a LightCycler™ (Roche Diagnostics, Mannheim, Germany). The amplification conditions comprised 95 °C for 10 s followed by 45 cycles of 95 °C for 5 s, 57 °C for 10 s and 72 °C for 10 s. Supplemental Table 1 shows the primer sequences. Amounts of target mRNA were normalized relative to that of *Actb*.

### Monitoring core body temperature

Mice were surgically implanted intra-abdominally with TempDisk TD-LAB data loggers (Labo Support Co. Ltd., Suita, Osaka, Japan) that were programmed to record body temperature (Tb) ± 0.1 °C every 15 min. Data obtained from each logger were analyzed using RhManager Ver.2.09 (KN Laboratories Inc., Ibaraki, Osaka, Japan) and hourly Tb values were averaged. We measured two-hour averaged Tb values between ZT13 and 15 during the experimental period to determine variations in peak Tb.

### Statistical analysis

All values are expressed as means ± SEM. Levels of mRNA expression in denervated and sham-operated mice fed with a normal diet or KD were statistically evaluated using a two-way analysis of variance (ANOVA) and the Tukey multiple comparison test using Excel-Toukei 2010 software (Social Survey Research Information Co. Ltd., Osaka, Japan). The value of Tb in mice fed with the KD and the mRNA levels in clock gene-mutant mice at corresponding ZT were compared between groups using Student’s *t*-test. The mRNA levels of *Slc2a25* in sciatic denervated mice and intact mice at corresponding ZT were compared between groups using one-way ANOVA. The effects of time on *Slc25a25* mRNA expression were determined by one-way ANOVA in denervated and *Clk/Clk* mice, and by Student’s *t*-test in M-*Bmal1* KO and G-*Bmal1* KO mice. The circadian amplitude of *Slc25a25* expression was set at half of the total peak-trough variation. Differences were considered significant at *P* < 0.05. Supplemental Tables 2–8 show the results of the statistical analysis.

## Electronic supplementary material

Supplementary Information
