# Supplementary material for: Ketogenic diet induces expression of the muscle circadian gene Slc25a25 via neural pathway that might be involved in muscle thermogenesis
Source: Sci Rep. 2017 Jun 6;7:2885. doi: 10.1038/s41598-017-03119-8 (PMC5460244; doi:10.1038/s41598-017-03119-8)
Supplement: Supplementary file 1 — Supplementary Information [file 41598_2017_3119_MOESM1_ESM.pdf]

Ketogenic diet induces expression of the muscle circadian gene *Slc25a25* via neural pathway that might be involved in muscle thermogenesis

Reiko Nakao[1], Shigeki Shimba[2], Katsutaka Oishi[1,3,4,*]

[1]Biological Clock Research Group, Biomedical Research Institute, National Institute of Advanced Industrial Science and Technology (AIST), Tsukuba, Ibaraki 305-8566, Japan

[2]Department of Health Science, School of Pharmacy, Nihon University, Funabashi, Chiba 274-8555, Japan

[3]Department of Applied Biological Science, Graduate School of Science and Technology, Tokyo University of Science, Noda, Chiba 278-8510, Japan

[4]Department of Computational and Medical Sciences, Graduate School of Frontier Sciences, The University of Tokyo, Kashiwa, Chiba 277-0882, Japan

[*]Corresponding author.

Supplementary Information

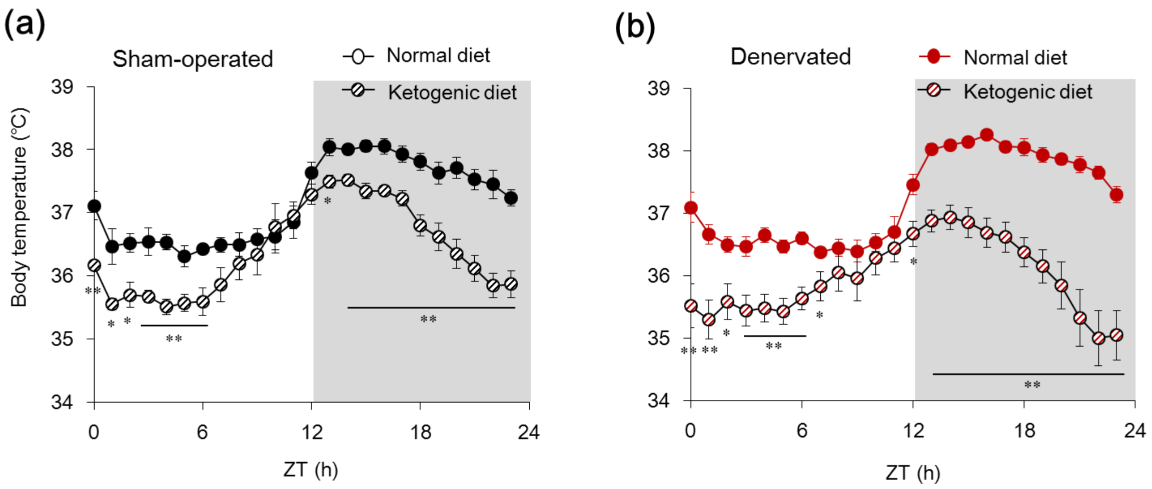

Supplemental Figure 1. Ketogenic diet reduces body temperature in sciatic denervated and sham-operated mice.

Core body temperature rhythms for 24 h in mice fed with ketogenic diet (KD) starting from 10 days after sham operation (a) or bilateral sciatic nerve transection (b). Hourly averaged values of body temperature at one day before (normal diet; ND) and after two weeks on KD in sham-operated (a) and sciatic denervated (b) mice. Gray shading indicates dark period. Data are expressed as means ± SEM (n = 5 - 7 per group). $^{*}P < 0.05$ and $^{**}P < 0.01$ for ND vs. KD. ZT; zeitgeber time. Supplemental Table 9 shows results of statistical analysis.

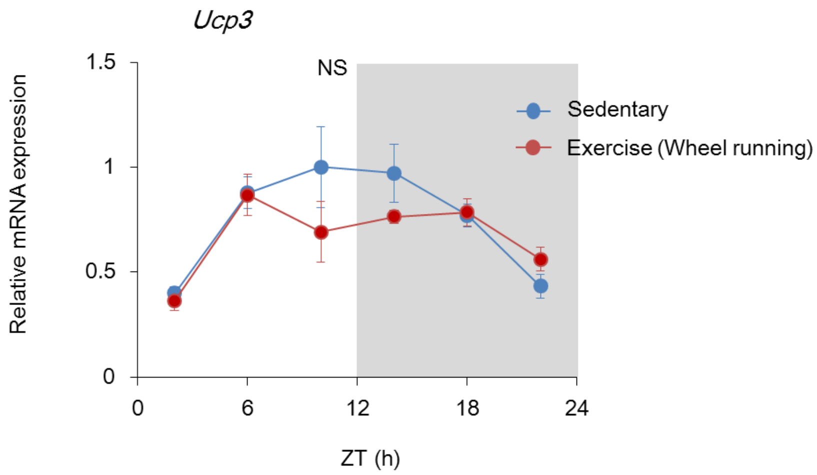

Supplemental Figure 2. Voluntary wheel running does not affect *Ucp3* mRNA expression in skeletal muscle.

Mice were individually housed in cages without running-wheels to mimic sedentary conditions or with running-wheels for four weeks. Gray shading indicates dark period. Data are means ± SEM (n = 4 - 5 per group). Maximal value for sedentary mice is expressed as 1.0. Supplemental Table 8 shows results of statistical analysis.

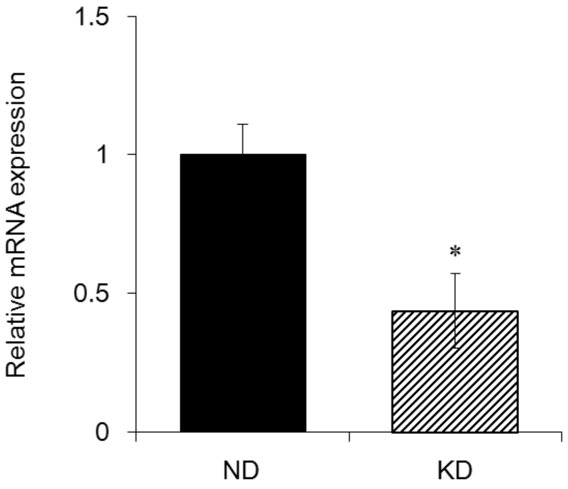

Supplemental Figure 3. Ketogenic diet decreases liver *Slc25a25* mRNA expression. Messenger RNA expression of *Slc25a25* in livers of mice fed with ketogenic (KD) or normal (ND) diet for 7 days. Data are expressed as means ± SEM (n = 4 per group). Values for mice given ND are expressed as 1.0. $^{*}P < 0.05$ ND vs. KD ($P = 0.018$ *t*-test).

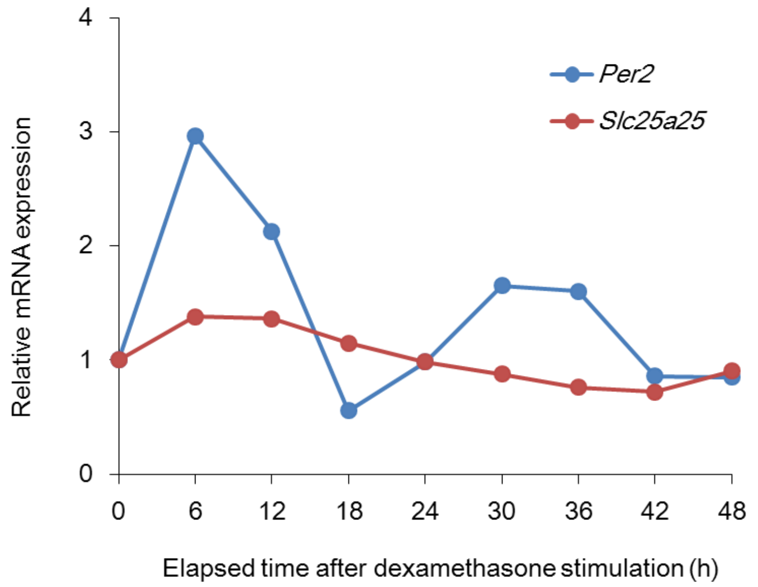

Supplemental Figure 4. ***Slc25a25*** **expression does not oscillate in C2C12 myotubes.**

Temporal expression profiles of *Slc25a25* and *Per2* mRNA in C2C12 myotubes. C2C12 cells were incubated in Dulbecco's modified Eagle's medium containing 10% fetal bovine serum (growth medium). Undifferentiated C2C12 cells were grown to confluence and then transferred to Dulbecco's modified Eagle's medium containing 2% horse serum (differentiation medium; changed every 48 h). The cells were stimulated 6 days later with 100 nM dexamethasone, and then collected every 6 h. Time 0 is expressed as 1.0.

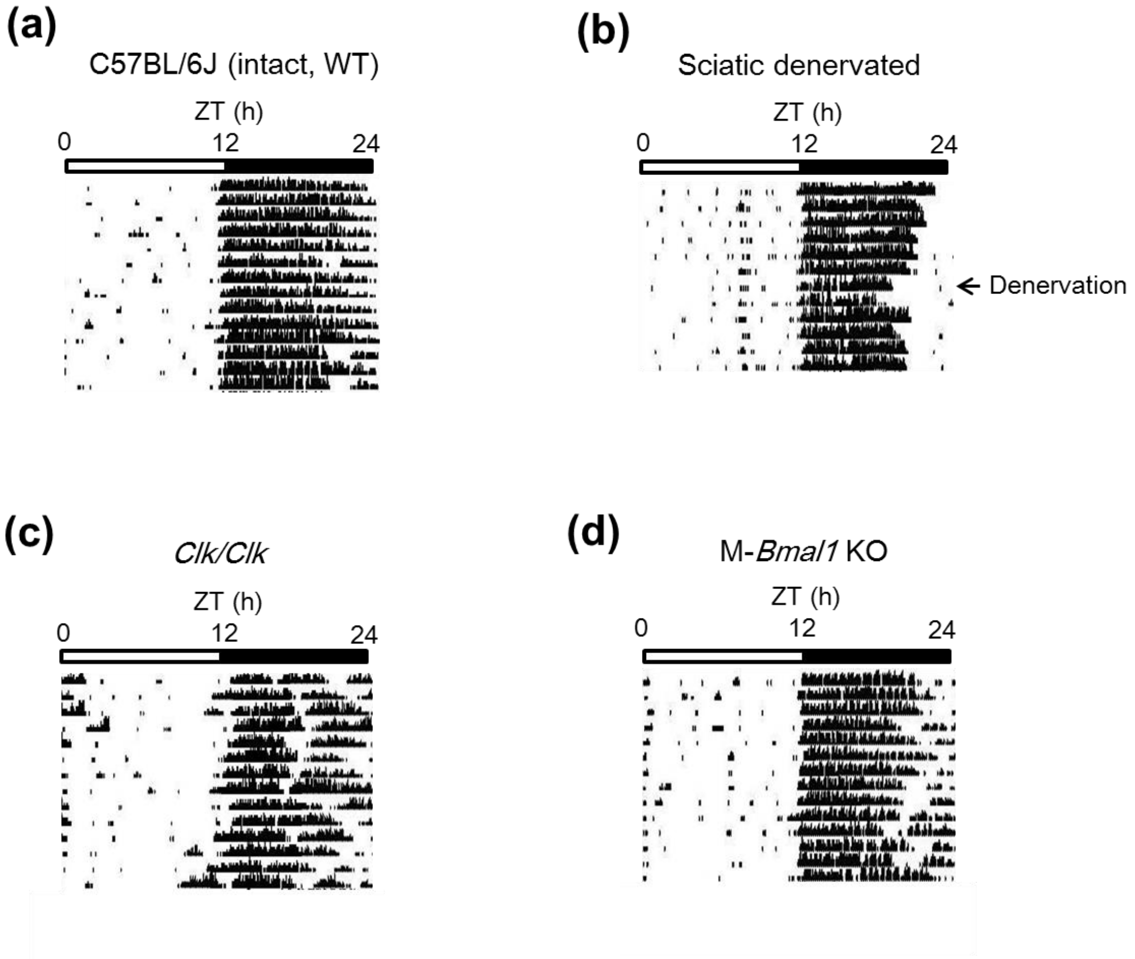

Supplemental Figure 5. Day/night locomotor activity is retained in denervated, *Clk/Clk*, and M-*Bmal1* KO mice.

Representative actograms of C57BL/6 (WT; a), denervated (b), *Clk/Clk* (c), and M-*Bmal1* KO (d) mice that were individually housed in cages with running-wheels. Wheel-running activity was continuously recorded using Chronobiology Kits (Stanford Software Systems, Stanford, CA). Locomotor activity was monitored at 5-min intervals and activity data are displayed as actograms as described[1]. Light/dark cycles are shown as white/black bars on each actogram, respectively.

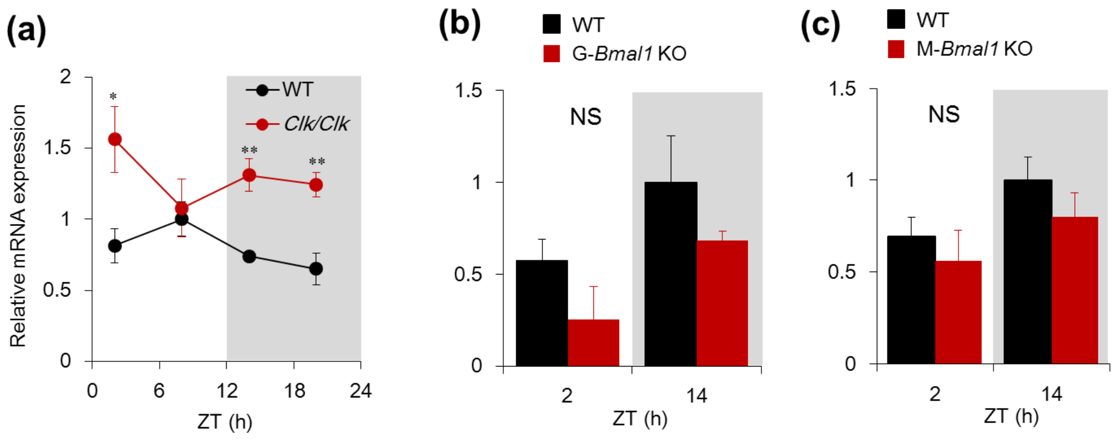

Supplemental Figure 6. *Clock* mutation induces *Slc25a25* expression in liver whereas *Bmal1* deletion has no effect.

Temporal expression profiles of *Slc25a25* mRNAs in liver of *Clock* mutant (a, *Clk/Clk*), global (b, G-*Bmal1* KO) or muscle-specific (c, M-*Bmal1* KO) *Bmal1* knockout mice. Data are expressed as means ± SEM (n = 4 - 5 per group). Maximal value for wild-type (WT) mice is expressed as 1.0. $^{*}P < 0.05$ and $^{**}P < 0.01$ for WT vs. mutant mice at corresponding zeitgeber time (ZT). Supplemental Table 3 shows results of statistical analysis.

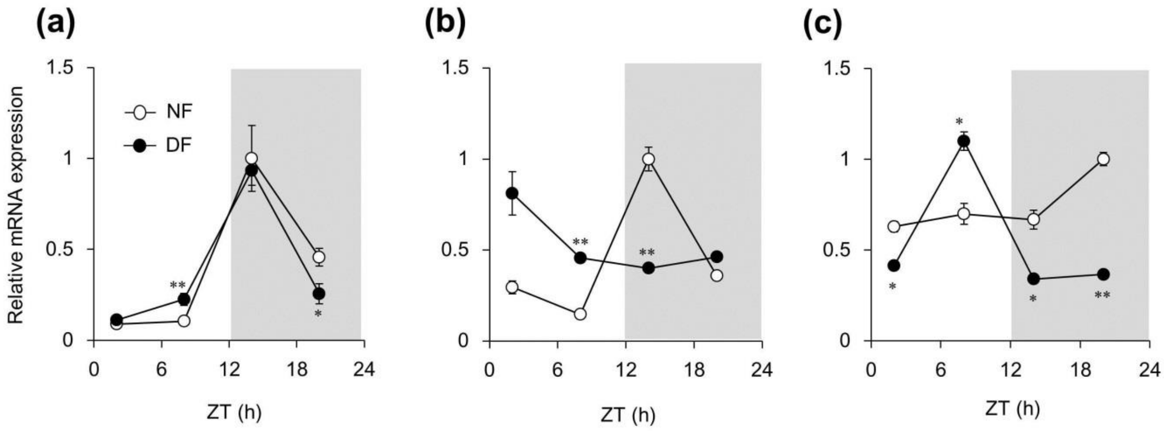

Supplemental Figure 7. Reversed feeding schedule shifts temporal expression profiles of *Slc25a25* mRNA in liver and white adipose tissue, but not in skeletal muscle.

Circadian expression of *Slc25a25* in skeletal muscle (a), liver (b) and white adipose tissue (WAT) (c) of mice fed during nighttime (NF; unfilled circles) or daytime (DF; filled circles). Time-imposed feeding was restricted as described[1]. Six-week-old male C57BL/6J mice (Japan SLC, Hamamatsu, Japan) were fed with a high-fat high-sucrose F2HFHSD diet (Oriental Yeast, Tokyo, Japan) *ad libitum* for two weeks under a 12-h light–12-h dark cycle. Mice were individually housed in cages with running wheels and then separated into groups that were fed only during sleep (ZT2-10; DF) or active (ZT14-22; NF) phases for one week. Data are shown as means ± SEM (n = 5). Maximal value for NF mice is expressed as 1.0. $^{*}P < 0.05$ and $^{**}P < 0.01$ for NF and DF mice at corresponding zeitgeber times (ZT). Supplemental Table 10 shows results of statistical analysis.

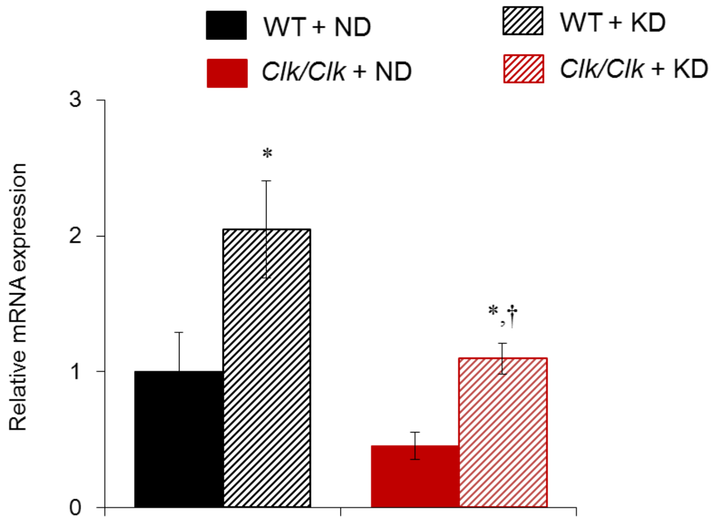

Supplemental Figure 8. Ketogenic diet induces *Slc25a25* mRNA expression in skeletal muscle of *Clk/Clk* mice.

Messenger RNA expression of *Slc25a25* in skeletal muscle of Clock mutant (*Clk/Clk*) or WT mice fed with ketogenic (KD) or normal (ND) diet for 7 days. Data are expressed as means ± SEM (n = 5 - 8 per group). Value for WT mice given ND is expressed as 1.0. $^{*}P < 0.001$ for ND vs. KD, $^{\dagger}P < 0.001$ for WT vs. *Clk/Clk*; $P = 0.332$ for interaction (two-way ANOVA).

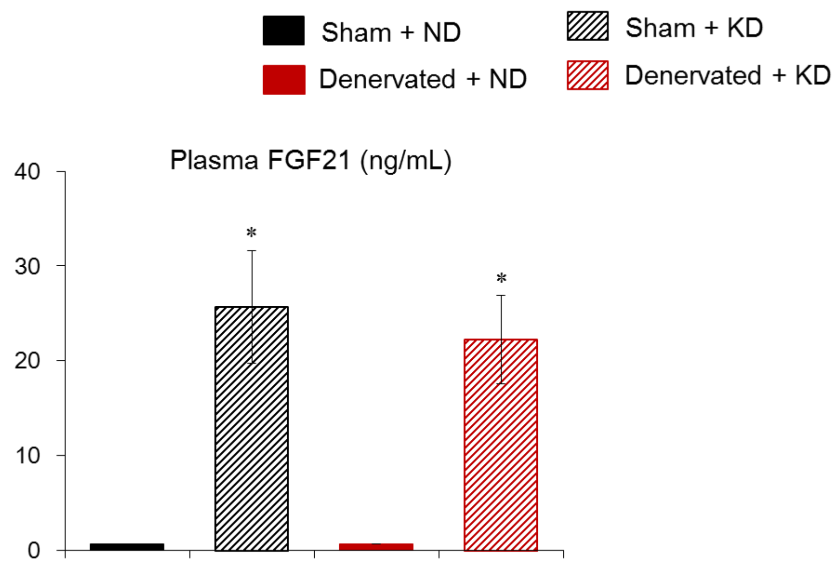

Supplemental Figure 9. Plasma FGF21 concentration does not significantly differ between sciatic denervated and sham-operated mice independently of diet. Mice were fed with ketogenic (KD) or normal (ND) diet for 7 days starting from 10 days after sciatic denervation or sham-operation. Blood collected in EDTA-coated tubes was immediately separated by centrifugation for 15 min at 5800 × *g* and then plasma was stored at −80°C. Plasma concentration of FGF21 was measured using mouse-/rat-specific FGF21 ELISA (BioVendor Inc., Karasek, Czech Republic). Data are means ± SEM (n = 5 per group). $P = 0.662$, sham-operated vs. denervated mice; $P < 0.001$ for ND vs. KD; $P = 0.655$ for interaction (two-way ANOVA).

Supplemental Table 1. Primer sequences for real-time RT-PCR.

Gene	Forward primer sequence (5' to 3')	Reverse primer sequence (5' to 3')
Slc25a25	GGGTGTCAAGATCTCGGAACA	GTAGTCCCTCCACTCGTTCCA
Slc25a23	TTGATTGGCAGGAATGGCGAGAC	GTCAGGCATTCACCGATGTCCA
Slc25a24	TGCAGCAGGGGCTGCAAAGCCTG	CATAAATTCTTCAAAATCCAGCTTC
Ucp1	CTCAGGATTGGCCTCTACGACTC	TTGGTGTACATGGACATCGCA
Ucp2	CTGGGACAGCTGCCTGCATTG	GTGCGCACTAGCCCTTGACTC
Ucp3	GTATGCTGAAGATGGTGGCTC	CGGAGATTCCCGCAGTACCTG
Sln	GCTCCTCTTCAGGAAGTGAAG	TGGCCCCTCAGTATTGGTAGG
Pgc1a	GTAGGCCCAGGTACGACAGC	GCTCTTGCGGTATTCATCCC
Nr1d1	CCCTGGACTCCAATAACAACACA	GCCATTGGAGCTGTCACTGTAG
Cidea	ATCACAACTGGCCTGGTTACG	TACTACCCGGTGTCCATTTCT
Actb	CACACCTTCTACAATGAGCTGC	CATGATCTGGGTCATCTTTTCA

Supplemental Table 2. Results of one-way ANOVA of mRNA expression in skeletal muscles of mice after sciatic denervation.

ZT	*p*
2	0.075
6	0.747
10	0.423
14	< 0.001
18	0.025
22	0.555

Supplemental Table 3. Results of Student's *t*-test of mRNA expression in skeletal muscles and liver of clock gene mutant mice.

Mouse strain	Tissue	ZT	*p*
Clk/Clk	Skeletal muscle	2	0.193
		8	0.134
		14	0.050
		22	0.430
	Liver	2	0.021
		8	0.753
		14	0.001
		22	0.003
G-*Bmal1* KO	Skeletal muscle	2	0.171
		14	0.011
	Liver	2	0.159
		14	0.387
M-*Bmal1* KO	Skeletal muscle	2	0.132
		14	0.036
	Liver	2	0.561
		14	0.322

Supplemental Table 4. Results of two-way ANOVA of gene expression profiles in mice fed with ketogenic diet or normal diet after sciatic denervation or sham-operation.

Tissue	Gene	Denervated/ Sham-operated	Normal/ Ketogenic diet	Denervation × KD
Skeletal muscle	*Slc25a25*	< 0.001	0.009	0.009
	Slc25a23	0.555	0.357	0.940
	Slc25a24	< 0.001	0.033	0.423
	Sln	< 0.001	0.831	0.838
	Pgc1a	< 0.001	0.644	0.540
	Ucp2	0.855	0.186	0.048
	Ucp3	< 0.001	< 0.001	0.004
	Nr1d1	0.055	0.361	0.781
BAT	*Slc25a25*	0.689	0.064	0.358
	Ucp1	0.192	0.932	0.645
	Cidea	0.087	0.160	0.101
	Pgc1a	0.138	0.960	0.050
	Ucp2	0.847	0.012	0.414
	Ucp3	0.603	0.679	0.278

	Nr1d1	0.504	0.044*	0.751
WAT	*Slc25a25*	0.969	0.160	0.292
	Ucp1	0.317	0.022	0.295
	Cidea	0.297	0.043*	0.554
	Pgc1a	0.066	0.077	0.811

*Significantly different by ANOVA, but not in post-hoc analysis.

Supplemental Table 5. Results of Student's *t*-test of body temperature at corresponding ZT.

	Normal diet	Ketogenic diet
ZT	*p*	*p*
0	0.954	0.152
1	0.537	0.511
2	0.921	0.769
3	0.770	0.457
4	0.510	0.940
5	0.403	0.620
6	0.210	0.877
7	0.417	0.933
8	0.826	0.739
9	0.464	0.479
10	0.748	0.279
11	0.685	0.135
12	0.499	0.050
13	0.883	0.020
14	0.402	0.037

15	0.424	0.145
16	0.166	0.044
17	0.395	0.072
18	0.250	0.206
19	0.166	0.228
20	0.382	0.332
21	0.251	0.195
22	0.404	0.156
23	0.739	0.135

Supplemental Table 6. Results of Student's *t*-test of peak body temperature during experimental period.

Day	*p*
1	0.027
2	0.851
3	0.633
4	0.194
5[*]	0.050
6	0.998
7	0.090
8	0.409
9	0.031
10	0.256
11	0.320
12	0.668
13	0.739
14	0.461
15[†]	0.616
16	0.100

17	0.786
18	0.081
19	0.026
20	0.085
21	0.014
22	0.038
23	0.086
24	0.170
25	0.020
26	0.009
27	0.004
28	< 0.001

[*]Day of denervation or sham-operation; [†]First day of ketogenic diet.

Supplemental Table 7. Results of Student's *t*-test of mRNA expression in skeletal muscle of adult and aged mice.

Gene	p
Slc25a25	0.030
Sln	< 0.001
Pgc1a	0.212
Ucp3	0.610

Supplemental Table 8. Results of Student's *t*-test of mRNA expression in skeletal muscles of mice housed with or without running wheel.

Gene	ZT	*p*
Slc25a25	2	0.195
	6	0.544
	10	0.012
	14	0.280
	18	0.107
	22	0.334
Ucp3	2	0.568
	6	0.935
	10	0.246
	14	0.194
	18	0.860
	22	0.150

Supplemental Table 9. Results of Student's *t*-test of body temperature at corresponding ZT.

	Sham-operated	Denervated
ZT	*p*	*p*
0	0.004	0.003
1	0.015	0.002
2	0.010	0.012
3	0.006	0.004
4	< 0.001	0.001
5	0.009	0.001
6	0.007	0.001
7	0.062	0.045
8	0.383	0.256
9	0.510	0.306
10	0.735	0.420
11	0.769	0.438
12	0.201	0.011
13	0.010	< 0.001
14	0.003	< 0.001

15	0.002	< 0.001
16	0.001	< 0.001
17	0.005	< 0.001
18	0.002	< 0.001
19	0.006	< 0.001
20	0.001	< 0.001
21	0.001	< 0.001
22	0.001	< 0.001
23	0.001	< 0.001

Supplemental Table 10. Results of Student's *t*-test of Slc25a25 mRNA expression in mice with time-imposed restricted feeding.

Tissue	ZT	*p*
Skeletal muscle	2	0.237
	8	0.009
	14	0.747
	22	0.025
Liver	2	0.100
	8	< 0.001
	14	0.004
	22	0.104
WAT	2	0.028
	8	0.047
	14	0.027
	22	< 0.001

References

1 Yasumoto, Y. *et al.* Short-term feeding at the wrong time is sufficient to desynchronize peripheral clocks and induce obesity with hyperphagia, physical inactivity and metabolic disorders in mice. *Metabolism* **65**, 714-727 (2016).
